# Malaria Epidemic and Drug Resistance, Djibouti

**DOI:** 10.3201/eid1102.040108

**Published:** 2005-02

**Authors:** Christophe Rogier, Bruno Pradines, H. Bogreau, Jean-Louis Koeck, Mohamed-Ali Kamil, Odile Mercereau-Puijalon

**Affiliations:** * IMTSSA-IFR48, Marseille, France;; † Centre Hospitalier des Armées Bouffard, Djibouti;; ‡ HIA R. Piquet, Bordeaux, France;; § Ministry of Health, Djibouti;; ¶ Institut Pasteur, Paris, France

**Keywords:** malaria, Plasmodium falciparum, Disease Outbreaks, Drug Resistance, genotype, merozoite surface protein 1, merozoite surface protein 2, dihydrofolate reductase, dihydropteroate synthase, chloroquine, dispatch

## Abstract

Analysis of *Plasmodium falciparum* isolates collected before, during, and after a 1999 malaria epidemic in Djibouti shows that, despite a high prevalence of resistance to chloroquine, the epidemic cannot be attributed to a sudden increase in drug resistance of local parasite populations.

From March to June 1999, an epidemic of *Plasmodium falciparum* malaria affecting all age groups spread in the city of Djibouti, Horn of Africa, an area with low and irregular transmission. Since the 1970s, autochthonous cases of malaria have been reported among the local population, but their incidence is usually low (*1*). *Anopheles arabiensis*, the main malaria vector in the city (*2**,**3*), has been found since the 1970s, possibly from Ethiopia (*1**,**4*). The focused distribution and the specificity of the breeding sites allowed a control strategy based on treatment of the larval sites with a larvivorous autochthonous fish, complemented with pinpoint use of bacterial toxins (*3*). Unfortunately, malaria control activities were progressively decreased so that, since the mid-1990s, vector control activity has been reduced to irregular insecticide indoor or outdoor spraying. Djiboutians frequently travel, and the Djibouti-Ethiopian railway has been suspected to be an effective route for propagating malaria parasites (*5*). Although some chloroquine treatment failures were reported in Djibouti in 1990 (*6*), most persons with *P. falciparum* were treated by chloroquine or quinine at the beginning of the 2000s, including during the 1999 epidemics. To determine whether this epidemic was associated with temporary changes in environmental conditions or to importation of new (virulent) or resistant *P. falciparum* strains, we investigated *P. falciparum* population diversity before, during, and after the outbreak and analyzed in vitro susceptibility profiles to a panel of antimalarials during the epidemics.

## The Study

The study was conducted at the Centre Hospitalier des Armées Bouffard, a French military hospital in Djibouti serving military and civilian natives from the entire city, and at other public health facilities of Djibouti. From 1997 to 2002, clinical malaria in the hospital shows the same temporal fluctuations as in dispensaries in the city (Figure). The incidence of patients with *P. falciparum* malaria admitted to the hospital increased >10-fold from March to May 1999 compared with the same period in 1997, 1998, and 2000–2002. In contrast, the number of admissions, consultations at the outpatient clinic, or blood counts performed for other causes than fever did not vary over the same period. The meteorologic station of the international airport of Djibouti recorded heavy rainfall the month before the epidemic. However, similar rainfall in 1997 or autumn 1999 was not followed by such a dramatic increase in malaria incidence in the ensuing months (Figure). When annual averages were compared, no particular variations in minimal or maximal mean air temperatures were found to occur during the months preceding the epidemic.

**Figure:**
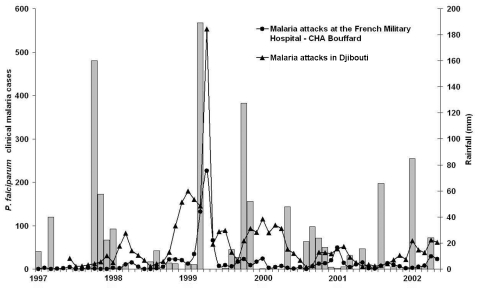
Rainfall (bars) and monthly incidence of *Plasmodium falciparum* clinical malaria cases (curve) at the French Military Hospital – CHA Bouffard (circle) and in the dispensaries of the city (Department of Epidemiology and Public Hygiene. Triangle), Djibouti city, January 1997–May 2002.

Forty-six blood samples were collected from September 14 to December 31, 1998 (period 1), 61 from April 12 to April 30, 1999 (period 2), and 32 from March 15 to May 15, 2002 (period 3), from patients with *P. falciparum* clinical cases who had not travelled outside the city of Djibouti during the preceding month and declared not having taken any antimalarial drug before the blood sampling. The study was cleared by the Djibouti Ministry of Health. Informed oral consent was obtained from patients before blood collection. Venous blood was collected before treatment administration in Vacutainer EDTA tubes (Becton Dickinson, Rutherford, NJ, USA). Thin blood smears were stained with an RAL kit (Réactifs RAL, Paris, France). Parasitemia was expressed as the proportion of *P. falciparum*–infected erythrocytes. Aliquots of freshly collected blood were kept at –20°C until DNA extraction.

*P. falciparum* genetic diversity was investigated by using *msp1* and *msp2* encoding highly polymorphic loci from merozoite surface protein genes. *Msp1* and *msp2* were genotyped by using nested polymerase chain reaction (PCR), as described (*7*), except that family-specific fluorescent primers were used in the nested PCR for assignment to the K1-, Mad20-, or Ro33-type *msp1* family and to the 3D7- or FC27-type *msp2* family. Fragment length was analyzed by the Genescan technology. Approximately 50% of the blood samples contained multiple *msp1* or *msp2* genotypes. The mean multiplicity of infection, i.e., the number of genotypes present in the blood sample, was ≈1.5 concurrent *P. falciparum* infections per person, with a decreasing tendency over the study period (Table 1). For each locus, multi-infection cases were excluded from analysis of genetic diversity. We identified 9 *msp1* alleles in 83 isolates and 17 *msp2* alleles in 108 isolates. The genetic diversity estimated by the unbiased expected heterozygocity (*8*), i.e., the probability that 2 randomly chosen genotypes are different in the sample, before, during, and after the 1999 outbreak was 0.79 (n = 23), 0.37 (n = 39), and 0.64 (n = 21) at the *msp1* locus and 0.83 (n = 31), 0.34 (n = 47) and 0.63 (n = 30) at the *msp2* locus, respectively. During the epidemic, Ro33-131 accounted for 79% of the *msp1* allele and FC27-408 accounted for 81% of the *msp2* alleles. Both alleles were present before and after the epidemic but with a much lower prevalence. They accounted for 26% of the *msp1* and 35% of the *msp2* alleles in 1998 and 14% of the *msp1* and 10% of the *msp2* alleles in 2002 (Table 2).

**Table 1:** Multiplicity of infections deduced from *msp1* and *msp2* genotyping and frequency (%) of the *Pfdhfr* (codons 51, 59, and 108), *Pfdhps* (codons 436, 437, and 540), and *Pfcrt* (codon 76) genotypes

Locus	Period 1 1998 (N = 46)	Period 2 1999 (N = 61)	Period 3 2002 (N = 32)	Total (N = 139)
*msp1*				
Mean multiplicity	1.6	1.5	1.3	
SD*	0.7	0.7	0.5	
No. of multiple infections (%)	23 (50)	22 (36)	11 (34)	56 (40)
*msp2†*				
Mean multiplicity	1.4	1.2	1.1	
SD	0.7	0.4	0.4	
No. of multiple infections (%)	14 (31)	12 (20)	2 (6)	28 (21)
*msp1* and *msp2*				
Mean multiplicity	1.8	1.6	1.4	
SD	0.7	0.7	0.6	
No. of multiple infections (%)	28 (61)	29 (48)	12 (38)	69 (50)
*Pf dhfr*				
Codon 51				
(Wildtype) N	38 (83)	60 (98)	15 (50)	113 (83)
N & I	3 (6)	0	0	3 (2)
I	5 (11)	1 (2)	15 (50)	21 (15)
Not genotyped	-	-	2	2
Codon 59				
(Wildtype) C	43 (94)	61 (100)	29 (97)	133 (97)
C & R	1 (2)	0	0	1 (1)
R	2 (4)	0	1 (3)	3 (2)
Not genotyped	-	-	2	2
Codon 108				
(Wildtype) S	37 (81)	60 (98)	16 (50)	113 (81)
S & N	2 (4)	0	0	2 (2)
N	7 (15)	1 (2)	16 (50)	24 (17)
*Pf dhps*				
Codon 436				
(Wildtype) S	46 (100)	52 (93)	29 (94)	127 (95)
F	0	1 (2)	0	1 (1)
A	0	3 (5)	2 (6)	5 (4)
Not genotyped	–	5	1	6
Codon 437				
(Wildtype) A	45 (98)	54 (96)	19 (61)	118 (89)
G	1 (2)	2 (4)	12 (39)	15 (11)
Not genotyped	–	5	1	6
Codon 540				
(Wildtype) K	44 (96)	60 (98)	19 (59)	123 (88)
K & E	1 (2)	0	0	1 (1)
E	1 (2)	1 (2)	13 (41)	15 (11)
*Pf crt*				
Codon 76				
(Wildtype) K	1 (2)	1 (2)	1 (3)	7 (5)
K & T	3 (7)	2 (3)	2 (6)	3 (2)
T	42 (91)	58 (95)	29 (91)	129 (93)

*SD, standard deviation; The genotypes at the *Pf dhfr*, *Pf dhps*, and *Pf crt* locus refer to the one-letter symbolized amino acids coded by the codons. A, Alanine; C, Cysteine; F, Phenylalanine; G, Glycine; I, Isoleucine; K, Lysine; N, Asparagine; R, Arginine; S, Serine; T, Threonine. †*msp2* multiplicity estimated on 45 and 59 samples in 1998 and 1999, respectively.

**Table 2:** Distribution of *msp1* and *msp2* alleles by allelic families and fragment size (in base pair) among Djibouti isolates with only 1 allele detected by locus*

Locus	Allelic families	Allele (base pair)	1998 (%)	1999 (%)	2002 (%)
*msp1*					
	K1	129	4.3	2.6	14.3
		203	0.0	2.6	0.0
	Mad 20	166	4.3	0.0	0.0
		184	34.8	2.6	57.1
		193	0.0	7.7	0.0
		202	21.7	5.1	14.3
		237	4.3	0.0	0.0
		241	4.3	0.0	0.0
	Ro 33	131	26.1	79.5	14.3
*msp2*					
	3D7	221	9.7	2.1	0.0
		226	0.0	2.1	0.0
		248	16.1	0.0	60.0
		253	0.0	2.1	0.0
		261	0.0	2.1	0.0
		275	0.0	10.6	0.0
		282	3.2	0.0	6.7
		284	0.0	0.0	3.3
		308	3.2	0.0	0.0
		346	0.0	0.0	3.3
		366	3.2	0.0	0.0
		371	0.0	0.0	3.3
	FC27	173	3.2	0.0	0.0
		373	6.5	0.0	6.7
		408	35.5	80.9	10.0
		444	3.2	0.0	0.0
		468	16.1	0.0	6.7

*Isolates collected in 1998 (*msp1*: n = 23; *msp2*: n = 31), 1999 (*msp1*: n = 39; *msp2*: n = 47), and 2002 (*msp1*: n = 21; *msp2*: n = 30).

To look for resistance-associated point mutations and haplotypes, the complete coding region of *Pfdhfr* (dihydrofolate reductase) and *Pfdhps* (dihydropteroate synthase) was amplified and sequenced (ABI 3100 Genetic Analyser, Applied Biosystems, Courtaboeuf, France) as described (*9*). We focused the analysis on point mutations of *Pfdhfr* codons 16, 51, 59, 108, and 164 and *Pfdhps* codons 436, 437, 540, 581, and 613, which have been associated with resistance to pyrimethamine and proguanil metabolite and to sulfadoxine, respectively (*10*). The prevalences of the *Pfdhfr* and *Pfdhps* mutations are shown in Table 1. No mutant was detected for *Pfdhfr* codons 16 and 164 and *Pfdhps* codon 581. A single isolate collected in period 2 harbored the *Pfdhps* A613S mutation. No isolate harbored the quintuple mutant haplotype (*Pfdhfr* S108N, N51I, and C59R and *Pfdhps* K540E and A437G) or the *Pfdhfr* C59R and *Pfdhps* K540E combination that predicts sulfadoxine-pyrimethamine clinical failure (*9*). One isolate containing at least 2 *P. falciparum* populations harbored 3 *Pfdhfr* mutations (S108N, N51I, and C59R) and the *Pfdhps* K540E mutation.

From 1998 to 1999, the frequency of isolates with mutated *Pfdhfr* codons 51, 59, and 108 decreased (not significantly), and *Pfdhps* allelic frequency did not differ significantly. The prevalence of isolates harboring the *Pfdhfr* N51I, *Pfdhfr* S108N, *Pfdhps* A437G, and *Pfdhps* K540E mutations increased from 1998–1999 to 2002 (Fisher exact test, p < 0.001 each). Presence of the chloroquine resistance–associated K76T mutation of *Pfcrt* (chloroquine-resistance transporter) (*11*) was analyzed by nested allele–specific PCR. Over the study period, 93% of the isolates harbored the *Pfcrt* K76T mutation (Table 1), without any significant temporal variation.

Twenty seven *P. falciparum* isolates collected during the 1999 epidemic with a 0.05%–5.0% parasitemia were transported at 4°C to our laboratory in Marseille, France, and analyzed for in vitro drug sensitivity by using an isotopic microtest (*12*). Among them, 93% were classified as resistant to chloroquine (Table 3). No isolate was resistant to amodiaquine. In vitro resistance was 4% for both pyrimethamine and cycloguanil.

**Table 3:** In vitro drug sensitivity of 27 *Plasmodium falciparum* isolates collected in Djibouti, 1999

Drugs	No isolates studied	Mean IC_50_*	95% confidence interval	Cut-off value	% resistant isolates
Chloroquine	27	326 nmol/L	224–474 nmol/L	>100 nmol/L	93
Amodiaquine	27	10.0 nmol/L	8.0–12.6 nmol/L	>80 nmol/L	0
Cycloguanil	24	13 nmol/L	8–21 nmol/L	>500 nmol/L	4
Pyrimethamine	25	69 nmol/L	41–117 nmol/L	>2,000 nmol/L	4

*The 50% inhibitory concentration (IC_50_) of chloroquine diphosphate, amodiaquine, pyrimethamine dihydrochloride, and cycloguanil, i.e., the drug concentration corresponding to 50% of the uptake of 3H-hypoxanthine by the parasites in drug-free control wells, was determined by nonlinear regression analysis of log-dose/response curves. Mean IC_50_ and proportion of resistant isolates according to cut-off values are indicated. Data were expressed as the geometric mean IC_50_ and 95% confidence intervals were calculated.

## Conclusions

Before and after the 1999 epidemic, *P. falciparum* genetic diversity in Djibouti was large, with ≈80% and 63% heterozygocity. This finding is somewhat surprising for an area where disease endemicity is low (*13*) and probably reflects importation of strains from neighboring areas such as Ethiopia or Somalia (*1**,**5*). *P. falciparum* genetic diversity was diminished during the epidemic, reflecting the circulation of a restricted number of strains during that period. Most of these strains harbored an *msp1* and *msp2* genotype that was detected before the epidemic. The prevalence of *Pfcrt*, *Pfdhfr*, and *Pfdhps* mutant genotypes did not vary significantly from 1998 to 1999. Thus, our data do not support the hypothesis of a sudden increase in the drug resistance of the local *P. falciparum* population as causing the epidemic. Our data are also not consistent with massive invasion by a single strain/genotype but rather suggest expansion during the epidemic of a few strains that were already prevalent. Further genotyping is needed to establish how many strains were circulating and their possible origin. What could have caused this sudden amplification? One possibility is a temporary increase in vector density. Unfortunately, no vectors were captured at that time, and this hypothesis is difficult to explore retrospectively.

The low prevalence of *Pfdhfr* and *Pfdhps* resistance mutations in 1998 and 1999 and of proguanil or pyrimethamine in vitro resistance in 1999 may explain the very low incidence of clinical malaria among the French soldiers stationed in Djibouti who were taking chloroquine-proguanil chemoprophylaxis. However, the sharp increase of *Pfdhfr* and *Pfdhps* resistance mutations observed in 2002 threatens sulfadoxine-pyrimethamine efficacy in the near future, even more so since the limited acquired immunity is unlikely to contribute to sustained drug efficacy (*14*). Molecular and in vitro assays point to a very high prevalence of chloroquine resistance. This finding calls for an urgent in vivo assessment of the antimalarials presently used in Djibouti in order to consider a rapid change in first-line treatment policy.

## References

[1] Carteron B, Morvan D, Rhodain F. Le problème de l'endémie palustre dans la République de Djibouti. Med Trop (Mars). 1978;38:837–46. 364244

[2] Shidrawi GR. Rapport sur une visite en République de Djibouti du 14 janvier au 11 février 1982. OMS/EM/MAL/190. Geneva: World Health Organization; 1982.

[3] Louis JP, Albert JP. Le paludisme en République de Djibouti. Stratégie de contrôle par la lutte antilarvaire biologique: Poissons larvivores autochtones (*Aphanius dispar*) et toxines batériennes. Med Trop (Mars). 1988;48:127–31. 3043137

[4] Courtois D, Mouchet J. Etude des populations de culicides en TFAI. Med Trop (Mars). 1970;30:837–46. 4396438

[5] Fox E, Bouloumie J, Olson JG, Tible D, Lluberas M, Shakib SO, *Plasmodium falciparum* voyage en train d'Ethiopie à Djibouti. Med Trop (Mars). 1991;51:185–9. 1895918

[6] Rodier GR, Parra JP, Kamil M, Chakib SO, Cope SE. Recurrence and emergence of infectious diseases in Djibouti city. Bull World Health Organ. 1995;73:755–9. 8907768 PMC2486693

[7] Zwetyenga J, Rogier C, Tall A, Fontenille D, Snounou G, Trape Jf, No influence of age on infection complexity and allelic distribution in *Plasmodium falciparum* infections in Ndiop, a Senegalese village with seasonal, mesoendemic malaria. Am J Trop Med Hyg. 1998;59:726–35. 9840589 10.4269/ajtmh.1998.59.726

[8] Nei M. Estimation of average heterozygosity and genetic distance from small number of individuals. Genetics. 1978;89:583–90. 17248844 10.1093/genetics/89.3.583 PMC1213855

[9] Kublin JG, Dzinjalamala FK, Kamwendo DD, Malkin EM, Cortese JF, Martino LM, Molecular markers for failure of sulfadoxine-pyrimethamine and chlorproguanil-dapsone treatment of *Plasmodium falciparum* malaria. J Infect Dis. 2002;185:380–8. 10.1086/338566 11807721

[10] Reeder JC, Rieckmann KH, Genton B, Lorry K, Wines B, Cowman AF. Point mutations in the dihydrofolate reductase and dihydropteroate synthetase genes and in vitro susceptibility to pyrimethamine and cycloguanil of *Plasmodium falciparum* isolates from Papua New Guinea. Am J Trop Med Hyg. 1996;55:209–13. 8780462 10.4269/ajtmh.1996.55.209

[11] Djimde A, Doumbo OK, Cortese JF, Kayentao K, Doumbo S, Diourte Y, A molecular marker for chloroquine-resistant *falciparum* malaria. N Engl J Med. 2001;344:257–63. 10.1056/NEJM200101253440403 11172152

[12] Pradines B, Rogier C, Fusai T, Tall A, Trape JF, Doury JC. In vitro activity of artemether and its relationship to other standard antimalarial drugs against West African isolates. Am J Trop Med Hyg. 1998;58:354–7. 9546418 10.4269/ajtmh.1998.58.354

[13] Anderson TJ, Haubold B, Williams JT, Estrada-Franco JG, Richardson L, Mollinedo R, Microsatellite markers reveal a spectrum of population structures in the malaria parasite *Plasmodium falciparum.* Mol Biol Evol. 2000;17:1467–82. 11018154 10.1093/oxfordjournals.molbev.a026247

[14] Plowe CV, Kublin JG, Dzinjalamala FK, Kamwendo DS, Mukadam RA, Chimpeni P, Sustained clinical efficacy of sulfadoxine-pyrimethamine for uncomplicated falciparum malaria in Malawi after 10 years as first line treatment: five year prospective study. BMJ. 2004;328:545–8. 10.1136/bmj.37977.653750.EE 14757706 PMC381042

